# Changes in Gene Expression Foreshadow Diet-Induced Obesity in Genetically Identical Mice

**DOI:** 10.1371/journal.pgen.0020081

**Published:** 2006-05-26

**Authors:** Robert A Koza, Larissa Nikonova, Jessica Hogan, Jong-Seop Rim, Tamra Mendoza, Christopher Faulk, Jihad Skaf, Leslie P Kozak

**Affiliations:** 1 Pennington Biomedical Research Center, Baton Rouge, Louisiana, United States of America; 2 Applied Biosystems, Foster City, California, United States of America; Stanford University, United States of America

## Abstract

High phenotypic variation in diet-induced obesity in male C57BL/6J inbred mice suggests a molecular model to investigate non-genetic mechanisms of obesity. Feeding mice a high-fat diet beginning at 8 wk of age resulted in a 4-fold difference in adiposity. The phenotypes of mice characteristic of high or low gainers were evident by 6 wk of age, when mice were still on a low-fat diet; they were amplified after being switched to the high-fat diet and persisted even after the obesogenic protocol was interrupted with a calorically restricted, low-fat chow diet. Accordingly, susceptibility to diet-induced obesity in genetically identical mice is a stable phenotype that can be detected in mice shortly after weaning. Chronologically, differences in adiposity preceded those of feeding efficiency and food intake, suggesting that observed difference in leptin secretion is a factor in determining phenotypes related to food intake. Gene expression analyses of adipose tissue and hypothalamus from mice with low and high weight gain, by microarray and qRT-PCR, showed major changes in the expression of genes of Wnt signaling and tissue re-modeling in adipose tissue. In particular, elevated expression of SFRP5, an inhibitor of Wnt signaling, the imprinted gene MEST and BMP3 may be causally linked to fat mass expansion, since differences in gene expression observed in biopsies of epididymal fat at 7 wk of age (before the high-fat diet) correlated with adiposity after 8 wk on a high-fat diet. We propose that C57BL/6J mice have the phenotypic characteristics suitable for a model to investigate epigenetic mechanisms within adipose tissue that underlie diet-induced obesity.

## Synopsis

Genetic models to explain the obesity epidemic are inadequate because the emergence of this epidemic over the past 30 y has been too rapid to allow for the appearance of new mutant genes. The authors show that diet-induced obesity among genetically identical mice is characterized by highly variable and stable phenotypes that are established in mice early in life, even before they become exposed to an obesogenic environment. Furthermore, strong associations occur between susceptibility to obesity and the expression of genes implicated in processes that regulate cellular development. Previous studies have shown that abnormal regulation of such genes by epigenetic mechanisms is linked with the development of cancer. Epigenetic mechanisms involve chemical processes that change chromatin structure and gene expression without changing the genetic code. Accordingly, epigenetic modifications of gene structure through nutritional and physiological stress provide mechanisms for inducing obesity that are independent of new mutations to the genome. Experimental models based upon genetically identical mice provide powerful tools for identifying epigenetic and environmental mechanisms causing obesity and other chronic diseases.

## Introduction

Obesity is a multifactorial disease in which inherited allelic variation, together with environmental variation, determines the predisposition of an individual to developing the disease. Although the evidence in support of a genetic component to the development of obesity is overwhelming [1–3], and a number of promising candidate genes are being tested as underlying causes of obesity [4], it remains difficult to quantify the genetic contribution to the obesity epidemic during the past 25 y, a period too short for the accumulation of additional obesogenic alleles. Twin studies indicate that the contribution of heritability to the obese phenotype may be as high as 70% [1,5]; however, this estimate includes allelic variation as well as genetic influences that are dependent upon a particular environment. Genomic and environmental variables are probably not independent, but gene-environmental interactions unique to each individual will determine the obese phenotype. Indeed it has been proposed that interactions between obesity genotypes and an obesogenic environment will synergistically increase the frequency of obesity [6]. Therefore, determining how allelic and environment variations interact to determine obesity phenotypes are critical for an understanding of the obesity epidemic.

Although we understand little regarding the interactions between genes and the environment that are associated with the development of obesity, the findings that some inbred strains of mice are susceptible to obesity when fed a high-fat diet, whereas others are resistant, clearly indicate that certain combinations of alleles are more obesogenic than others [7,8]. In addition, over- or under-expression of selective genes can have major effects on diet-induced obesity, but little or no effect when animals are fed a low-fat diet [9–11]. To develop a model system to discover the basis for environmental components of obesity, we have taken advantage of diet-induced obesity in highly inbred C57BL/6J (B6) mice. Male mice of this inbred strain are more susceptible to obesity when fed a high-fat diet [12] than other strains such as 129 mice [13]. However, we and others have observed large variations in adiposity and diabetes among the individual B6 mice, even when maintained under seemingly identical environmental conditions [14–16]. We hypothesized that in the absence of allelic variation among the animals, phenotypic variation would be determined by either unstable stochastic mechanisms, or by stable epigenetic modifications of genes of energy balance. We have developed an experimental strategy to determine variations in obesity phenotypes in a large population of B6 mice and to analyze patterns of gene expression by microarray analysis and quantitative RT-PCR (qRT-PCR) to identify metabolic pathways associated with extreme adiposity phenotypes.

In this paper, we present evidence for highly variable, but stable, obesity phenotypes among B6 mice by 6 wk of age, even before they are fed a high-fat diet, indicating that some mice are destined to be high gainers, while others from the same litter are to become low gainers. An analysis of the energy cost of weight gain suggests that variation in both metabolic efficiency and food intake are determinants of the obesity phenotype. The microarray analysis of gene expression in adipose tissue from high and low gainers suggests that the Wnt signaling pathway and genes associated with vascularization and tissue remodeling are major regulatory points controlling differences in adipose tissue expansion and that some of these genes are differentially expressed even before mice are fed a high-fat diet.

## Results

### Non-Genetic Variation in Body Weight and Composition

**Figure 1:**
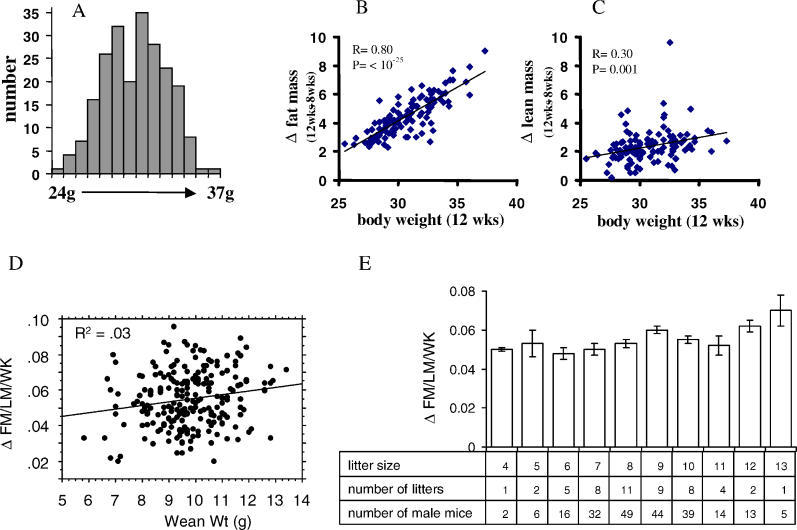
Establishing Variations in Adiposity in Diet-Induced Obesity in Male C57BL/6J Mice Frequency distribution of body weight in 219 mice at 12 wk of age, after being fed a high-fat diet for 4 wk. (A) Regression analysis between the change in body weight and fat mass (B) and change in body weight and lean body mass (C) from 8 and 12 wk of age as determined by NMR in 112 mice. (D) Regression analysis between mouse weight at weaning and the percent change of the ratio of fat mass (FM) to lean mass (LM) per week, *p* = .0105, *n* = 220. Changes in the FM/LM ratio per wk were calculated from 8–12 wk of age or from 8–14 wk of age. (E) Average percent change of the ratio of FM/LM per week for the various litter sizes, *p* = .0406, *n* = 220 (ANOVA).

Genetically identical 8-wk-old male B6 mice developed a highly variable obesity after being fed a diet containing 58 Kcal % saturated fat (Figure 1). Body weights of 219 mice fed a high-fat diet for 4 wk were distributed in a bell-shaped curve ranging from 24–37 g (Figure 1A)**.** Based upon determinations of body composition using nuclear magnetic resonance (NMR), body weight variation was primarily caused by changes in adiposity (Figure 1B) with a much smaller, though significant contribution, from lean body mass (Figure 1C). This morphometric evidence shows that variability in fat mass among individual B6 mice at the extreme ends of the distribution leads to about a 4-fold difference in fat mass after only 4 wk on a high-fat diet. Critical to the experimental design to identify factors determining diet-induced obesity was the necessity to minimize variation not related to the effects of a high-fat diet, such as that coming from variation in litter size with its subsequent effects on the size of pups at weaning, since it has been shown that litter size can have strong effects on development of adiposity [17–19]. Regression analysis of body weight at weaning versus changes in adiposity (fat mass/lean mass, ΔFM/LM) and analysis of variance (ANOVA) of litter size versus changes in adiposity (ΔFM/LM) at 12 wk (Figure 1D and 1E) barely reached significance and the *R* ^2^ indicated that the variation in wean weight contributed about 3 % to the variance in adiposity. Breeding and dietary conditions in our facility produced progeny with low variability in body weight (at 3 wk of age, the 112 mice in Figure 1B had a mean and standard deviation for body weight of 10.99 ± 1.23 g). Thus the variation in body weight in 12-wk-old mice, following 4 wk on a high-fat diet, was not significantly influenced by differences in litter size and body weights of pups prior to weaning.

### The Stability of the Adiposity Phenotype

**Figure 2:**
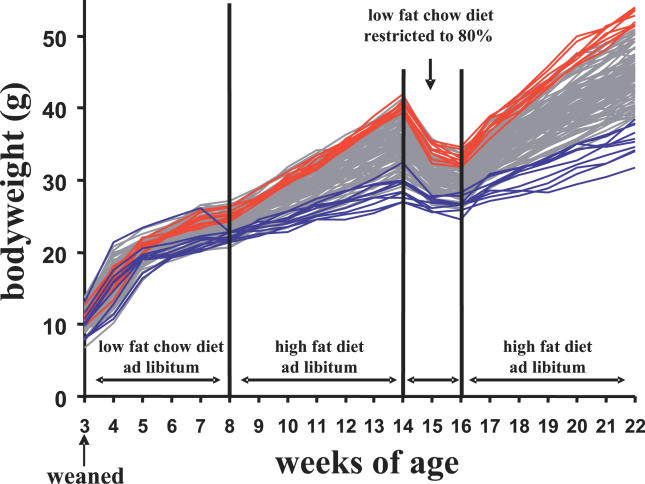
Stability of the Adiposity Phenotype in B6 Mice Male mice (*n* = 107) were fed a low-fat chow diet (Picolab 5053) from weaning to 8 wk of age, a high-fat diet (Research Diets D12331) from 8–14 wk, then the low-fat chow diet, restricted to 80% of the amount consumed from wk 7–8, during wk 15 and16, and finally, the high-fat diet for wk 17–22. Mice were weighed weekly except during the food restriction period when body weights were measured daily until they had stabilized under these conditions. Food intake was measured weekly starting from wk 7. At the end of wk 8, 14, 16, and 22, the body composition of each mouse was analyzed by NMR. The body weight curves of mice at the upper and lower 10% of the frequency distribution at 22 wk of age are plotted in red and blue.

The observed variation could be the result of stochastic, random variation in the activity of any of a large number of metabolic pathways associated with the development of obesity. Alternatively, the obesity phenotype in individual mice could be fixed, and once acquired, stable, a necessary condition for a specific epigenetic mechanism. To determine the stability of the obesity phenotype 107 male B6 mice were fed a low-fat chow diet ad libitum from weaning until they were 8 wk of age and then a high-fat diet ad libitum for 6 wk, until they were 14 wk of age to induce obesity (Figure 2). At the end of the 14^th^ wk, we returned the mice to the low-fat chow diet (restricted to 80% of the amount consumed by each mouse from wk 7–8) until they reach a new low plateau. We expected that body weights would return to that of mice not fed a high-fat diet, which would lie between 22–26 g. Although the body weights of all mice declined during this 2-wk period of restricted low-fat feeding, it is striking that all mice did not arrive at the same plateau; rather, their weights were higher, between 23–35 g and distributed over a range proportional to that observed before caloric restriction. The metabolism and behavior of the high gainer mice had adapted to maintain a higher body weight and adiposity even when they were fed a low-fat restricted diet, reminiscent of the behavior of obese humans who defend their body weights when fed a restricted diet [20,21]. At the beginning of the 17^th^ wk the mice were again fed the high-fat diet and they immediately responded by increasing their adiposity at a rate commensurate with that observed during the first exposure to the high-fat diet. The rate of gain of the top 10 % was 2.2 times greater than that of the lower 10 % from the 8^th^ to the 14^th^ wk (2.54 ± 0.04 versus 1.15 ± 0.09 g/wk) and also 2.2 times greater from the 16^th^ to the 22^nd^ wk (3.29 ± 0.05 versus 1.51 ± 0.11g/wk). We conclude that the obese phenotypes of B6 mice fed a high-fat diet are stable and consistent with a mechanism that causes a permanent change in energy metabolism.

We have shown in the previous section that litter size and body weights at weaning (3 wk of age) did not correlate with body weights at 12 wk of age, after 4 wk on a high-fat diet (Figure 1D). However, the correlation of body weights of mice at 8 wk of age fed a low-fat diet to the change in fat mass from 8–12 wk of age was highly significant (*R* = 0.364; P = 7.99 × 10^−05^). This evidence together with inspection of the growth curves in Figure 2 show that differences between high and low gainers have been established even before the mice have received a high-fat diet. The high-fat diet accentuates differences in energy balance that already appear to exist among progeny. The potentiality to express these phenotypic differences must have been established earlier in development, but were not evident from body weight phenotypes at weaning.

### The Cost of Weight Gain or Metabolic Efficiency

**Figure 3:**
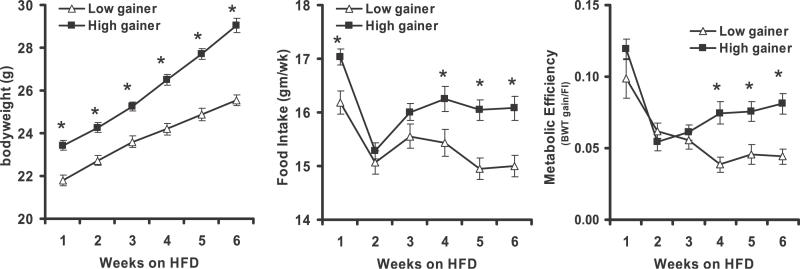
Chronology of Body Weight, Food Intake, and Metabolic Efficiency of Mice with Food Intake Restricted to an Amount Equivalent to That Consumed by 80% of Experimental Mice Twenty mice were in each group. Differences in phenotype between groups are marked by an asterisk (*) at a significance level of *p* < 0.01

The energy cost of weight gain is a complex process that depends on many factors. The major variable in our dietary studies has been the fat content of diets 5053 and D12331, containing 12 and 58 kcal % fat respectively, while protein content at 23.6 and 16.5 kcal %, respectively, is within a normal range that does not stimulate thermogenesis [22]. The early emergence and stability of phenotypic differences observed in Figure 2, even during caloric restriction, suggests that diet per se is not the underlying mechanism for the variability in obesity. To address this issue further it was necessary to modify our standard protocol to achieve minimal food spillage, which can be considerable with B6 mice fed ad libitum. The feeding protocol was based upon first restricting the available food to one pellet, but still in excess and available ad libitum, that was given to the mice in the late afternoon of each day beginning at wk 7 when the mice were on a low-fat diet. At the beginning of the 8^th^ wk when the high-fat diet was started, the amount of diet, still in one pellet, given to the mice was titrated to a level where approximately 80% of the mice left a portion of the food pellet in the hopper. By the end of the first week this amount was estimated at 2.7 g and held constant at that amount for the remainder of the experiment. The amount of food consumed for each mouse was estimated daily and food consumption is presented as the amount consumed per mouse per week for the top 20 high gainers and bottom 20 low gainers (Figure 3). Body weights for the high gainers were significantly different from low gainers after 1 wk on a high-fat diet and they become steadily greater during the following weeks. In contrast, food intake and the feeding efficiency, calculated by dividing the body weight gained for each week by the food consumed for that week, was not significantly different between the high and low gainers until the 4^th^ wk of high-fat feeding. Thus, although differences in food intake appear to contribute to the long term variation in obesity among mice, they did not appear to be a determinant in establishing the initial differences in adiposity. Important to physiological mechanisms related to adiposity and food intake is the potential involvement of leptin. Plasma leptin levels (mean ± SD = 78.9 ± 29.9 ng/ml; range 27.6–168; *n* = 107) from mice in Figure 2 were strongly correlated with adiposity after 6 wk on a high-fat diet (*R* = 0.617; *p* = 1.42 × 10^−12^).

### Molecular Correlates Associated with Variation in Adiposity

We have used microarray analysis of gene expression to obtain a global view of the pathways of adipogenesis by which genetically identical mice of the same age and sex and fed the same diet differentially expand their fat mass. Using the Applied Biosystems (Foster City, California, United States) Expression Array System, as described in the Materials and Methods, replicate analysis of a pool of RNAs from high and low gainers was performed with RNA from both the hypothalamus and inguinal fat depots. Analysis of expression in inguinal fat revealed 792 genes that were over-expressed in high gainers and 1,110 that were over-expressed in low gainers at a significance level of *p* < 0.01 and with a cutoff of 1.5 for over-expression in high and low gainers, respectively. The difference in expression for high gainers/low gainers for the complete data set ranged from +23.9 (mesoderm specific transcript, MEST) to −14.3 (lymphocyte antigen 74). In contrast to the large number of genes with significant differences in expression in inguinal fat, only eight genes showed altered expression in the microarray analysis of the hypothalamus. However, none of the differences in microarray expression of these genes could be validated by qRT-PCR with analysis of 20 individual high gainers and 20 low gainers. In addition, another ten genes, which have been localized to defined regions in the hypothalamus and known to be involved in food intake, showed no significant differences in expression when analyzed by qRT-PCR. (See Table S1 for data on gene expression in the hypothalamus.) Thus, we conclude that variations in mRNA levels for components of signaling pathways for food intake in the hypothalamus are not significantly variable between high and low gainers.

To interpret the large number of gene targets showing significant differences in gene expression in adipose tissue the following strategy was developed:

The list of genes was inspected manually to identify those with known functions in adipocyte biology. These could be associated with the mature adipocyte, preadipocytes, or vascular tissue.

Literature searches were performed on genes with highly significant differences to establish associations with adipose tissue biology.

Individual adipose tissue samples from 20 high gainers and 20 low gainers were analyzed together with RNA samples isolated from the stromal vascular and mature adipocyte fractions on a custom-designed quantitative TaqMan® Low Density Array carrying 48 genes on the primary list (Table 1). The analysis of the different fat pads was useful to filter out genes expressed in contaminating cells, for example, lymphatic tissue in inguinal fat and the epididymus in epididymal fat. A gene that was only expressed in one depot and in only a few of the samples in an inconsistent pattern was eliminated from further consideration.

**Table 1:**
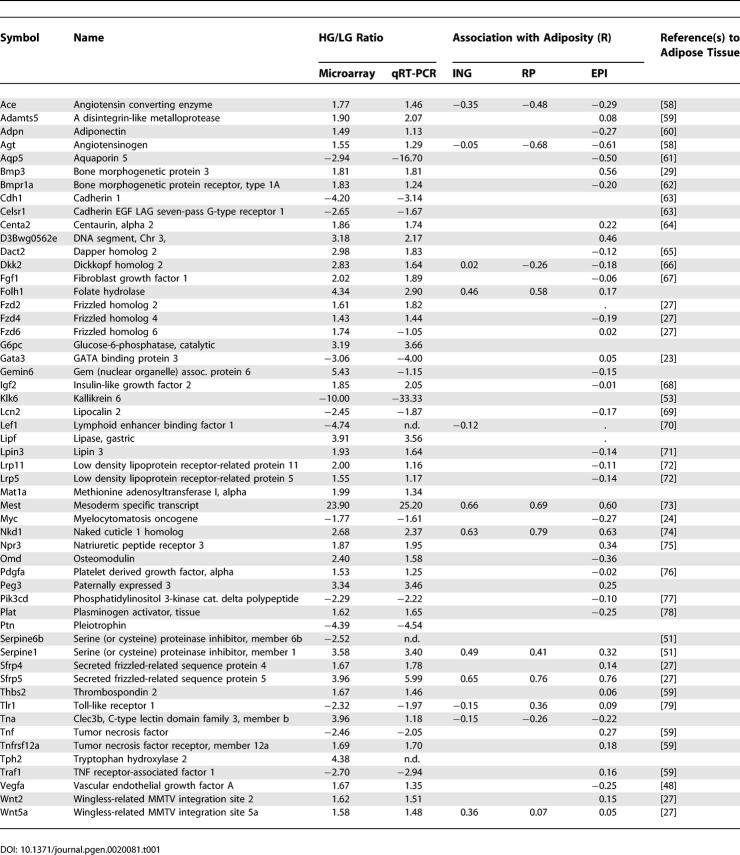
Validation of Microarray Gene Expression Data

Genes with highly significant differences, but with no published evidence for involvement in adipose biology, were not pursued further at this time.

### Transcription Factors

Transcription factors associated with adiposity generally showed no changes in expression that reach significance between high gainers and low gainers. Exceptions included Stat 4, Lef1 (a target of Wnt signaling), E2F2, (microarray data), and the three factors GATA3, Lipin3, and Myc. The expression of Lef1 in the stromal vascular fraction, its absence from retroperitoneal fat and epididymal fat and from the inguinal fat depots of many animals suggested that Lef1 is expressed in lymphocytes that were trapped within the inguinal fat depot. Similarly, GATA3 and Myc have been implicated in the regulation of proliferation of preadipocytes [23,24] and were considered viable candidates, but they did not survive following analysis of other fat depots. Consequently, none of the genes on the final list of candidates were transcription factors.

### Biomarkers of the Adipocyte and Energy Metabolism

Similar to transcription factors strongly implicated in adipogenesis, the biomarkers of the adipocyte and genes encoding enzymes of energy metabolism showed remarkably little change in expression. The largest and most significant change was in the 1.78-fold increase in leptin (unpublished data), a response expected from increased adiposity. Consistent with this increase in mRNA levels, leptin levels in the serum of mice in the experiment described in Figure 2 correlated significantly with body weight (*R* = 0.617; *p* = 1.42 × 10^−12^ ; *n* = 107). The β3 adrenergic receptor was expected to have reduced levels of expression in high gainers, but the reduction was not significant, possibly because 4 wk of a high-fat diet did not achieve sufficiently high levels of adiposity to down regulate its expression [25]. Significantly reduced β3-adrenergic receptor mRNA was observed in high gainers in a preliminary analysis of adipose tissue after 12 wk on a high-fat diet (unpublished data).

### Wnt Signaling

**Figure 4:**
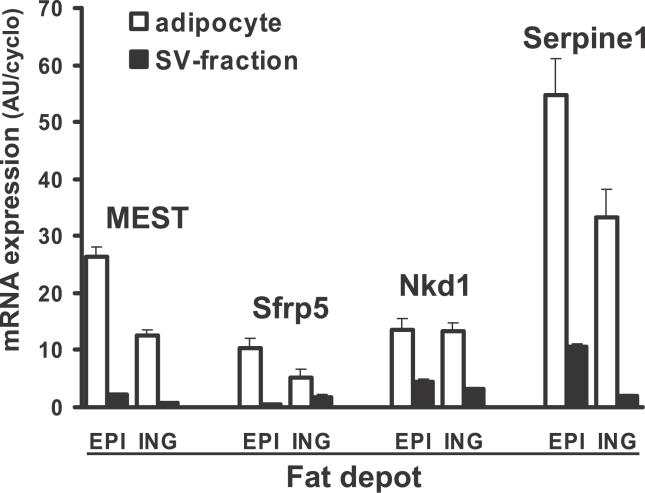
Comparison of the Expression of Four Genes in the Mature Adipocyte and Stromal Vascular Fraction of the Epididymal and Inguinal Fat Pads Isolated from Four Pools of RNA Derived from Tissue Isolated from Eight Mice Each pool consisted of equal amounts of RNA from two mice. All comparisons between adipocyte and stromal vascular fractions were significant (*p* < 0.01) except for ING fat SFRP5 (*p* = 0.09). EPI = epididymal; ING = Inguinal

**Figure 5:**
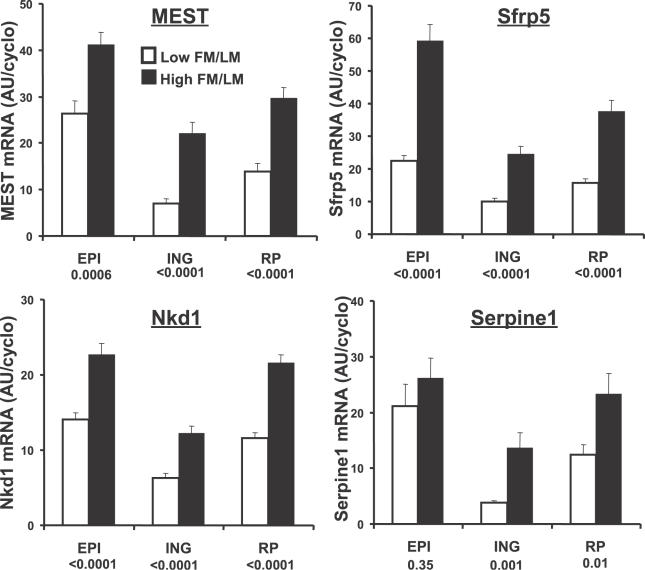
Over-Expression of Four Genes in High Gainer Mice in Two Peritoneal Fat Depots and One Subcutaneous Fat Depot Illustrates that the Mechanism Leading to Over-Expression of These Genes Occurs in All Fat Depots Statistical significance was calculated by ANOVA. Twenty mice were present in each group.

**Figure 6:**
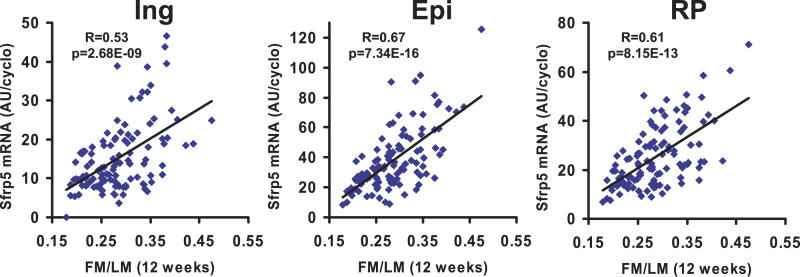
Positive Correlations between SFRP5 and Adiposity Regression analysis between SFRP5 mRNA levels and adiposity as estimated by the ratio of FM to LM in ING, EPI, and RP fat from 112 Mice Described in Figure 1B. FM = Fat Mass; LM = Lean Mass; ING = Inguinal; EPI = Epididymal; RP = Retroperitoneal

**Figure 7:**
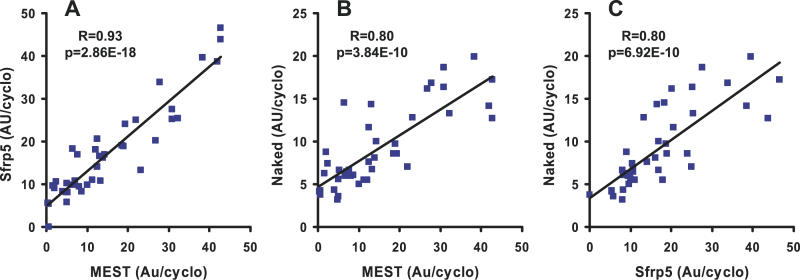
Coordinated Gene Expression Regression analyses of SFRP5, MEST, and Naked mRNA levels in RNA isolated from high and low gainer mice in inguinal fat depots suggest that regulation of this subset of genes share a common mechanism related to the degree of adiposity. Twenty mice were present in each group.

An extensive series of investigations have implicated Wnt signaling molecules, especially Wnt 10b, as agents that maintain the preadipocyte compartment and inhibit their differentiation into mature adipocytes [26,27]. Small changes were noted in the expression of some members of the Wnt family, but not Wnt 10b, nor in the levels of mRNA of their frizzled receptors; however, the reproducibility of changes in these genes were not confirmed by qRT-PCR of RNA samples from individual mice and multiple fat depots. On the other hand, increased levels of expression of two inhibitors for Wnt signaling, secreted frizzled related protein (SFRP) 5 and Naked1, were among the most significantly increased in expression in high gainers (Table 1). The range in expression for SFRP5 was 30–44-fold depending on the fat pad and that of Naked1 about 5 fold. SFRP5 and Naked1 were predominantly expressed in the adipocyte fraction (Figure 4). The expression of SFRP5, Naked1, MEST, and Serpine1 were all elevated in three different fat depots of high gainer mice (Figure 5). Regression analysis of SFRP5 to adiposity indicates that variation in epididymal fat SFRP5 is associated with as much as 45 % of the variance in adiposity (*R* = 0.67; Figure 6). In addition, the high correlations between SFRP5, Naked1, and MEST in inguinal fat depots suggest a mechanism of coordinated regulation of the expression of these genes in association with susceptibility to diet-induced obesity (Figure 7).

### Imprinted Genes

Four imprinted genes showed a highly significant induction in high gainers, particularly PEG1/MEST with a 23-fold increase in the adipocytes of high gainers compared to low gainers (Table 1). A detailed analysis of MEST expression is being prepared for an independent publication.

### Vascularization and Tissue Remodeling

Several genes with potential functions in the control of vascularization show highly significant differences in expression between high and low gainers. Of particular note Serpine1 or plasminogen activator inhibitor 1, known to be involved in vascularization and the control of adipogenesis, was over-expressed in the inguinal and retroperitoneal fat depots of high gainers (Figure 5). Angiotensinogen gene expression was elevated in visceral fat, but not subcutaneous fat as previously shown [28], but curiously the correlation between angiotensinogen mRNA levels in retroperitoneal and epididymal fat with adiposity among the 20 high gainers and 20 low gainers was negative. Bone morphogenetic protein 3 (BMP3), which is antagonistic to bone development [29], showed a strong association with increased adiposity.

### Molecular Correlates in the Pre-Obese State

**Figure 8:**
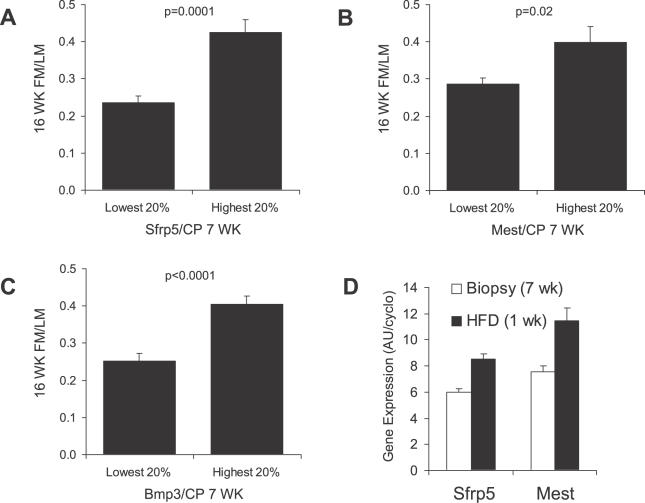
Analysis of mRNA Levels in Biopsies of Epididymal Fat Depots Taken from Mice 7 wk of Age prior to Feeding a High-Fat Diet Suggests That Changes in Gene Expression Exist before Mice Are Exposed to an Obesogenic Environment Mean adiposity (FM/LM) at 16 wk of age for the mice with the highest and lowest 20% expression of SFRP5 (A), MEST (B), and BMP3 (C) at 7 wk of age. SFRP5 and MEST were assayed by qRT-PCR and BMP3 by a TaqMan® Low Density Array. Each bar represents the mean FM/LM ± SEM for 12 mice. (D) Induction of SFRP5 and MEST mRNA levels after being fed a high-fat diet at 8 wk of age for 1 wk.

Inspection of the chronological development of obesity from weaning at 3 wk of age until 22 wk of age, during which a high-fat diet was fed from 8 wk of age for 14 wk (including a gap of 2 wk when mice received a restricted low-fat diet) indicates that mice destined to become high gainers were already beginning to show signs of increased adiposity at 6 wk of age even before they were fed a high-fat diet (Figure 2). In order to evaluate whether genes, which were found to be up-regulated in obese mice, showed altered levels of expression prior to being fed a high-fat diet, we performed an experiment in which biopsies of epididymal fat were taken at 7 wk of age and the mice were continued on the protocol of diet-induced obesity by feeding the high-fat diet from 8–16 wk of age. Gene expression in biopsied samples was determined using the TaqMan® Low Density Array described in Table 1 to validate changes in the high density microarray analysis (Figure 8). Only three genes, SFRP5, MEST, and BMP3, showed significant associations with the degree of adiposity present in the FM/LM index of the biopsied mice at 16 wk of age (Figure 8A–8C). Thus, variation in expression of selected genes implicated in diet-induced obesity was evident even before the mice were submitted to an obesogenic environment. Furthermore, it was found that a significant induction of SFRP5 and MEST mRNA occurred after only 1 wk on a high-fat diet (Figure 8D).

## Discussion

### Epigenetic Model of Obesity

The motive to explore the basis for highly variable obesity phenotypes periodically observed to occur within inbred strains of mice is that it intuitively suggests a model system to identify regulatory mechanisms underlying obesity that are independent of inherited mutations to the genome. If a stable, though variable, obesity phenotype is present among individual mice of a “normal” highly inbred strain, then it may reflect the workings of an epigenetic mechanism for obesity together with environmental and genetic mechanisms. Given the strong opinions on the genetic versus environmental basis for the obesity epidemic [30,31], a model that establishes an epigenetic contribution to obesity would be of significant value. A major focus of this study has been to establish rigorously the variable obesity phenotype in an inbred strain, since it had previously been based upon anecdotal observations of phenotypes of control mice in studies of transgenic and knockout models of obesity. The following evidence from our study is consistent with an epigenetic mechanism underlying the variable obesity phenotypes present among male B6 mice fed a high-fat diet: 1) a robust 4-fold range in the development of adiposity occurred in a population of B6 male mice within 4 wk of being fed a high-fat diet (Figure1); 2) the phenotypic differences were stable, as shown in Figure 2, and remained so even after the regimen of a high-fat diet fed ad libitum was interrupted by feeding a restricted low-fat chow diet to reduce their adiposity state; 3) variations in gene expression in 7-wk-old mice prior to the introduction of an obesogenic diet correlated with obesity phenotypes after 8 wk on a high-fat diet (Figure 8).

Our model to explore the epigenetic basis of diet-induced obesity is designed around a conscious effort to minimize malnutrition and stress. The variation in litter size or wean weight showed weak positive associations with diet-induced obesity in the adult that contributed approximately 3% to the variance in adiposity. In earlier stages of our study B6 dams were fed a diet with 6.5% fat rather than the 11% fed during later experiments and produced offspring with a wider range of wean weights, but even with these animals the association of wean weight with diet-induced obesity was always positive (unpublished data). In contrast to these almost insignificant associations between wean weights and adiposity, which were estimated by regression analysis to contribute only ~3% to the variance in adiposity after 4 wk on a high-fat diet, the association of adiposity with levels of SFRP5 mRNA was as high as 45% (Figure 6). A popular current model to investigate nutritionally determined epigenetic programming of obesity and type 2 diabetes has emerged from the fetal origins hypothesis of Barker, in which individuals exposed to malnutrition during in utero development have increased susceptibility to obesity when exposed to an obesogenic environment as an adult [32,33]. It is unclear whether the mechanisms determining variations in obesity in the well nourished animals of our studies have any relationship to those by which nutritional programming in the malnourished fetus predestines the individual by adaptation to become obese, since in contrast to our model the low-birth-weight individuals become more obese. However, it is not difficult to imagine how developmental genes, like SFRP5, BMP3, and MEST, could also be involved in determining variations in adipogenesis that are rooted in fetal malnutrition.

### Physiological Mechanisms

The keystone of this obesity model is the difference in adiposity that emerges following the feeding of a high-fat diet. However, variation in adiposity may not be the primary mechanism, rather changes in adiposity could also follow from differences in the caloric load and these could be determined by differences in food intake and/or in feeding efficiency. The latter could be particularly complex and could involve the processing of nutrients via the gastrointestinal tract, energy metabolism in the periphery, and the efficiency of fat storage. To obtain clues that implicate food intake or feeding efficiency, we have related the development of the adiposity among individual mice with changes in food intake and calculated feeding efficiency. The results suggest that differences in adiposity during the first 3 wk of the high-fat diet do not correlate with differences in either food intake or feeding efficiency. However, during the subsequent 4^th^, 5^th^, and 6^th^ wk both feeding efficiency and food intake were significantly correlated with differences in adiposity (Figure 3). Since it is known that B6 mice with diet-induced obesity develop resistance to peripheral leptin, as evidenced by the effects on food intake [34] and the high gainers in our experiment indeed have higher leptin levels than low gainers, the changes in food intake and feeding efficiency that follow the early differences in body weight could in part reflect the effects of leptin resistance. Elevated levels of leptin expression would stimulate leptin resistance, which in turn would lead to increased food intake and possible suppression of energy expenditure as evidenced by the increased feeding efficiency. Whatever the mechanism underlying differences in food intake, no differences in gene expression could be detected in the hypothalamus by microarray analysis and qRT-PCR. The experiments have largely focused on the phenotypes that emerge in the presence of an obesogenic environment, i.e. a high-fat diet; however, our evidence suggests that fundamental changes in the epigenetic status of the genome of individual animals may be established prior to the introduction of the high-fat diet. Recently studies indicating that variations in the leptin surge during the neonatal period can impact upon later susceptibility to weight gain on a high-fat diet suggest that stochastically determined variations in the leptin surge in B6 mice need to be evaluated [35]. However, even if such a leptin-based mechanism is involved in determining subsequent differences in susceptibility to diet-induced obesity, understanding the role of the developing adipose tissue as the source of the leptin surge remains a key factor.

Assuming that variation in adiposity is determined epigenetically; possibly by a mechanism involving chromatin methylation, when during development does this occur? If changes in the epigenetic state of the genome depend upon an obesogenic environment, then the post-natal period when adipose tissue is in its most active state of proliferation [36], and when the fat content of the neo-natal diet approaches 55 Kcal% [37] would be an intriguing period within which to search for establishing epigenetic modification of chromatin in adipocytes. Alternatively, epigenetic differences among individuals may be stochastically determined at any stage of development and the consequences of the variation become evident under the appropriate conditions, the most important of which appears to be the genetic background of the animal and a high-fat diet to induce gene expression. The presence of variations in the expression of SFRP5 and other genes in 7-wk-old mice suggests that modifications in chromatin structure that cause these variations among individual mice exist prior to the introduction of an obesogenic diet. However, it is unknown whether these epigenetic variations are actually affecting the SFRP5 gene or some upstream regulator.

### Molecular Mechanisms

Our finding that highly significant differences in expression occur for specific genes in metabolic pathways of adipogenesis establishes that the molecular mechanisms for the variation in obesity are not caused by random stochastic variation in gene expression among individual animals, since such random activations of gene expression would not lead to reproducible patterns of expression among individuals in a population. Rather, the microarray data and subsequent qRT-PCR analyses of high and low gainers suggest that highly significant differences in gene expression within specific molecular mechanisms of adipogenesis occur among a large fraction of the mice. Molecular pathways of adipogenesis have been extensively investigated during the past several years [38,39]; however, it is not known whether unique regulatory pathways exist that control the expansion of adipose tissue mass during environmentally-induced obesity. The analysis of data was based upon viewing the adipose tissue as a composite of three compartments: 1) the mature adipocyte, 2) the preadipocyte fraction, and 3) the vascular epithelium. Partial information on genes that were preferentially expressed in these compartments was obtained by analyzing expression in mature adipocytes and cells from the stromal vascular fraction. The complete gene expression database was searched to evaluate the behavior of genes that have previously been implicated in the three compartments of adipogenesis. In addition, an important feature of the analysis was to highlight not only genes with significant variation between high and low gainers, but to note the absence of significant changes in genes that are biomarkers of the adipocyte or have been strongly implicated in adipogenesis. Accordingly, one of the major initial insights from the gene expression data is the absence of changes between high and low gainers in the expression of genes encoding either the major transcription factors of adipogenesis or biomarkers of the adipocyte, both of which are robustly induced during in vivo adipogenesis and in cell culture models [39]. A few transcription factors such as GATA3, Lipin3, and Myc were variably expressed, but these may be more involved in gene expression of cells in the stromal vascular fraction, possibly preadipocytes.

### Wnt Signaling

The characterization of Wnt10b expression with over-expression and gene knockout models in cell culture and in vivo has provided strong evidence that the Wnt signaling pathway plays an important role in the differentiation of adipocytes from mesenchymal precursors [27,40]. Wnt signaling proceeds through the canonical pathway that stabilizes cytoplasmic levels of β-catenin by dephosphorylation and facilitates its translocation to the nucleus where it controls a program of target gene expression in preadipocytes that inhibits their progression to a mature adipocyte [41]. Gene targets of the transcription mechanism act to suppress PPARγ expression and activate genes of osteogenesis [36]. Several regulators of the Wnt pathway have been identified; some act by inhibiting the interaction of Wnt ligands with the frizzled receptors, these include members of the SFRP family; others like Dickkopf interfere with the formation of an active frizzled receptor-LRP complex; and Naked1 is a protein that interacts with Dishevelled to enhance the formation of the axin-APC-GSK3 complex that promotes phosphorylation of the β-catenin complex and its degradation by a proteasome-mediated pathway [42].

Although forced expression of Wnt10b and SFRP1 in 3T3-L1 cells and transgenic mice will inhibit or stimulate preadipocyte differentiation, respectively, there is very little evidence documenting the function of these components of the pathway during adipogenesis in vivo [26,43]. In other words, does Wnt10b play as central a role in the control of adipogenesis in vivo as suggested from the published studies or do other members of the pathway play additional complementary roles? We have presented evidence on significant changes in the expression of additional genes associated with Wnt signaling. Changes in expression were small for Wnt2 and Wnt5a and for frizzled receptors, and not detected for Wnt10b, the most thoroughly studied Wnt family member. While differences in expression for these molecules scarcely reached significance, four genes (SFRP5, Dapper, Dickkopf, and Naked1) with demonstrated functions as inhibitors of Wnt signaling were highly up-regulated in high gainers. A 30–40-fold variation in SFRP5 and 5-fold variation in Naked1 mRNA levels were highly correlated with indices of adiposity (*R* = 0.67; Figure 5 and 6), as well as with each other and MEST with *R* values ranging between 0.8 and 0.93 (Figure 7). These associations between gene expression and variable obesity phenotypes may merely indicate that the changes in expression of SFRP5, MEST, and other genes only result as a consequence of adiposity. To address this question we assessed whether these genes have altered levels of expression in the pre-obese state that correlate with susceptibility to obesity that occurs following exposure to an obesogenic environment. The results indicate that SFRP5, MEST, and BMP3, but not Naked1, are positively associated with the subsequent development of diet-induced obesity in 16 wk-old mice. In addition, the induction of SFRP5 and MEST mRNA levels after only 1 wk on a high-fat diet may indicate that these genes are highly sensitive to the obesogenic environment. These genes may be regulated by transcription factors that are responsive to ligands derived from the high-fat diet or to morphological/mechanical changes induced by the uptake of intracellular lipids when the high-fat diet is introduced. The importance of cytoskeletal-derived forces in the regulation of adipocyte differentiation was described over 20 y ago by Spiegelman and Ginty [44] and more recently McBeath et al [45] showed that a microenvironment that changes the shape of stem cells in culture will determine whether they differentiate into osteocytes or adipocytes. Given the reciprocal response of mesenchymal stem cells to proceed along either the osteogenesis or adipogenic pathways [46], the increased expression of BMP3 which has been shown to antagonize osteogenesis [29], is consistent with a role for BMP3 in adipogenesis.

The function of Wnt signaling in adipogenesis, as elucidated by the analysis of Wnt10b in 3T3-L1 cells, is based upon maintaining the preadipocyte compartment, where Wnt10b is exclusively expressed [27], through the control of cell proliferation and apoptosis. The function of Wnt signaling in diet-induced obesity is likely to be different, since SFRP5 is predominantly expressed in the mature adipocyte and Naked1 is expressed in both the mature adipocyte and stromal-vascular fractions. The importance of SFRP5 as a regulatory site in an epigenetic mechanism was recently shown with the demonstration that methylation and repression of the promoters for SFRP1, −2, −4, and −5 in colorectal tumor cells causes constitutive expression of Wnt signaling and enhances tumor promotion. Accordingly, similar DNA -methylation and/or histone modifications of the SFRP genes in mice during diet-induced obesity are possible. While the gene expression analyses of our study did not show significant differences in expression for the major transcription factors associated with adipogenesis, this obviously does not address a role for transcription mechanisms of adipogenesis involving protein phosphorylation or nuclear translocation that are surely involved in regulating fat mass expansion in diet-induced obesity.

### Vascularization and Tissue Remodeling

It is clear from recent studies that differentiation and expansion of adipose tissue mass are linked to mechanisms associated with angiogenesis and vascularization. The fat mass of the both genetic and diet-induced obesity of the mouse is drastically reduced by inhibitors of angiogenesis [47], an effect that is also observed in tissue culture models [48]. Plasminogen activator inhibitor 1 is elevated in both mouse and human obesity [49], and mice carrying target mutations to this gene have reduced obesity and improved insulin resistance [50,51]. Other studies have shown important roles for the Adamts disintegrin-protease system [52] and kallikrien serine proteases associated with the plasminogen cascade for the modeling of adipose tissue [53]. Therefore it is of considerable interest that variation of these genes between the high and low gainers are among the most robust differences we have observed (Table 1) and suggests an additional site for determining susceptibility to environmentally induce obesity.

In summary, we have shown that individuals within an inbred strain of mouse susceptible to diet-induced obesity have a large variation in stable obesity phenotypes. We also show that high gainers are more metabolically efficient in the conversion of caloric energy into body weight and eventually have greater food intake; this is likely a secondary consequence of early increases in fat mass and leptin production. Gene expression analysis identified two important aspects of adipocyte biology where variation in the adiposity could be achieved. One is in reversing the inhibition of adipogenesis from the mesenchymal cell compartment by Wnt signaling mechanisms through the methylation-sensitive SFRPs and BMP3, which have elevated expression in mice destined to become high gainers even before they are fed a high-fat diet. The other is in systems associated with vascularization and tissue remodeling, the modulation of which strongly influences obesity [47,51]. This research underscores how variation in energy balance in an inbred strain provides a model to explore epigenetic mechanisms that are as powerful as mutations to Mendelian genes in causing obesity. Parenthetically, it becomes obvious that if there is a background of stable variation in diet-induced obesity within in an inbred strain that exceeds the quantitative contribution of any single genetic locus, why it is so difficult to identify obesity quantitative trait loci. Finally, it is certain that the variable molecular and physiological obesity phenotypes we have described in this study represent the tip of the iceberg of a plethora of variable, stable somatically heritable phenotypes that are probably present within inbred strains and as shown for the Axin gene, epigenetic inheritance can be influenced by genetic background [54].

## Materials and Methods

### Animals.

C57BL/6J breeders were obtained from the Jackson Laboratory (Bar Harbor, Maine, United States). To minimize the genetic drift from the colonies at the Jackson Laboratory breeding stock was replaced every 2 y. All mice for the experiments were generated on site at the Pennington Biomedical Research Center. Animal rooms were maintained at 22–24 °C with a 12-h light/dark cycle. Breeders were housed in plastic pens with corn-cob bedding and fed a breeders diet 5015 ad libitum. From weaning until 8 wk of age mice were fed a low-fat chow diet 5053 ad libitum. (Composition of diets 5015 and 5053 are available from www.labdiet.com). At 8 wk of age mice were fed ad libitum a high saturated fat diet D12331 (Research Diets, New Brunswick, New Jersey, United States) for various periods of time as indicated by the experimental protocol. The effects of a diet high in unsaturated fat was tested with a diet formulated by Research Diets that was modeled after D12331 and contained 16.5 kcal% protein, 38.5 kcal% carbohydrate, and 45.0 kcal% fat (4.8% hydrogenated coconut oil and 40.1% corn oil). From weaning until 7 wk of age mice of the same sex were group housed (3–5 mice per pen) until 7 wk of age, at which time they were singly housed for the remainder of the experiment*.*

### Epididymal fat biopsy.

In 7-wk-old mice on a low-fat diet, the caudoventral half of the abdomen was shaved to remove hair and surgically prepped with three alternating scrubs of Nolvasan and 70% ethanol. An incision of the paramedian skin approximately 1.0–1.5 cm was made in the right or left caudal quadrant, followed by a 0.5-cm incision through the abdominal wall to access the epididymal fat depot. Thumb forceps were used to retract the epididymal fat and approximately 100 mg of adipose tissue excised. The remaining fat was replaced into the abdomen. The abdomen was closed in two layers. First the abdominal wall was surtured using 4–0 Vicryl, followed by wound clips to close the skin. Wound clips were removed 7–10 d post operatively. All animal experiments were approved by the Pennington Biomedical Research Center Institutional Animal Care and Use Committee in accordance with NIH guidelines.

### Phenotyping.

Adiposity was determined from body weights and measurements of body composition by NMR (Bruker, The Woodlands, Texas, United States). Energy content of weight gain is based upon an energy content of 30 kJ/g, if body composition is 60% fat and 45 kJ/g if 100% [22]. Food intake was determined on a weekly basis, unless otherwise indicated, by weighing the food remaining in the food hopper. Energy content of the high-fat diet (D12331) is 23.29 kJ/g. Animals that spilled excessive food were not included in the food intake data. At the conclusion of a particular experiment, tissues were quickly removed and frozen in liquid nitrogen and stored at −80 °C for subsequent preparation of total RNA. Total RNA was isolated from adipose tissue and hypothalamus using TRI Reagent (Molecular Research Center Inc. Cincinnati, Ohio, United States) with modifications to remove DNA using the Qiagen RNAeasy columns and DNaseI Kit (Qiagen, Valencia, California, United States). RNA was stored at −70 °C in RNase-free H_2_O supplemented with the RNase inhibitor Superasin (Ambion, Austin, Texas, United States) per manufacturers directions. Quality and quantity of RNA was determined using either UV spectrophotometry and agarose gel visualization of intact RNA or by using the Agilent Bioanalyzer as per manufacturers procedures (Agilent Technologies, Palo Alto, California, United States). Quantitative RT-PCR (qRT-PCR) using TaqMan probes and primers or Sybr green (Applied Biosystems, Foster City, California, United States) was performed essentially as described previously [55,56] except standard curves were generated using pooled RNA from individual samples within each experiment. Probe and primer sequences used to perform the analyses are available upon request. Validation of the expression levels of a large number genes identified by microarray analysis (see below) was also performed by a custom-designed TaqMan® Low Density Array (Applied Biosystems, Foster City, California, United States).

### Microarray analysis.

Gene expression profiles were generated using Applied Biosystems Mouse Genome Survey Microarray. Each microarray contains approximately 34,000 features that include a set of about 1,000 controls. Each microarray uses 32,996 probes to interrogate 32,381 curated genes representing 44,498 transcripts.

Prior to amplification and labeling, the quality of total RNA isolated from adipose tissue was determined using the Agilent 2100 Bioanalyzer. The RNA Integrity Number (RIN) of the RNA ranged from 8.8–9.1. One μg of total RNA was used to transcribe DIG-labeled cRNA using Applied Biosystems Chemiluminescent RT-IVT Kit v2.0. Microarray hybridization (using 20 μg of fragented, DIG-labeled cRNA and three microarrays per sample), processing, chemiluminescence detection, imaging, auto gridding, and image analysis was done according to Applied Biosystems protocols and the 1700 Chemiluminescent Microarray Analyzer Software v. 1.0.3. Signal intensities across microarrays were normalized using the quantile-quantile method (http://www.bioconductor.org). Features with a signal/noise values ≥ 3 and quality flag values < 5,000 were considered “detected” and were subjected to ANOVA analysis with *p* value of 0.01 using Spotfire DecisionSite Software (Spotfire, Somerville, Massachusetts, United States). The lists of differentially expressed genes with a cutoff of 1.5 for over-expression in high and low gainers were then classified using PANTHER ([57], http://www.panther.appliedbiosystems.com).

## Supporting Information

Table S1 Microarray Analysis of the Hypothalamus of High and Low Gainer Mice (131 KB PDF)

### Accession Numbers

Microarray experiments, described according to MIAME guidelines, have been deposited in NCBI's Gene Expression Omnibus (GEO, http://www.ncbi.nlm.nih.gov/geo) repository with accession numbers GSE4697 and GSE4692 for the hypothalamus and inguinal fat, respectively.

## Abbreviations

ANOVA: analysis of variance
BMP3: bone morphogenetic protein 3
EPI: epididymal
FM: fat mass
ING: inguinal
LM: lean mass
MEST: mesoderm specific transcript
NMR: nuclear magnetic resonance
qRT-PCR: quantitative reverse-transcriptase polymerase chain reaction
RP: retroperitoneal
SFRP: secreted frizzled related protein

## References

[pgen-0020081-b001] Bouchard C, Tremblay A, Despres JP, Nadeau A, Lupien PJ (1990). The response to long-term overfeeding in identical twins. N Engl J Med.

[pgen-0020081-b002] Allison DB, Kaprio J, Korkeila M, Koskenvuo M, Neale MC (1996). The heritability of body mass index among an international sample of monozygotic twins reared apart. Int J Obes Relat Metab Disord.

[pgen-0020081-b003] Maes HH, Neale MC, Eaves LJ (1997). Genetic and environmental factors in relative body weight and human adiposity. Behav Genet.

[pgen-0020081-b004] Perusse L, Rankinen T, Zuberi A, Chagnon YC, Weisnagel SJ (2005). The human obesity gene map: The 2004 update. Obes Res.

[pgen-0020081-b005] Stunkard AJ, Harris JR, Pedersen NL, McClearn GE (1990). The body-mass index of twins who have been reared apart. N Engl J Med.

[pgen-0020081-b006] Barsh GS, Farooqi IS, O'Rahilly S (2000). Genetics of body-weight regulation. Nature.

[pgen-0020081-b007] Surwit RS, Feinglos MN, Rodin J, Sutherland A, Petro AE (1995). Differential effects of fat and sucrose on the development of obesity and diabetes in C57BL/6J and A/J mice. Metabolism.

[pgen-0020081-b008] Hu CC, Qing K, Chen Y (2004). Diet-induced changes in stearoyl-CoA desaturase 1 expression in obesity-prone and -resistant mice. Obes Res.

[pgen-0020081-b009] Kopecky J, Hodny Z, Rossmeisl M, Syrovy I, Kozak LP (1996). Reduction of dietary obesity in aP2-Ucp transgenic mice: Physiology and adipose tissue distribution. Am J Physiol.

[pgen-0020081-b010] Liu X, Rossmeisl M, McClaine J, Riachi M, Harper ME (2003). Paradoxical resistance to diet-induced obesity in UCP1-deficient mice. J Clin Invest.

[pgen-0020081-b011] Bachman ES, Dhillon H, Zhang CY, Cinti S, Bianco AC (2002). betaAR signaling required for diet-induced thermogenesis and obesity resistance. Science.

[pgen-0020081-b012] Surwit RS, Kuhn CM, Cochrane C, McCubbin JA, Feinglos MN (1988). Diet-induced type II diabetes in C57BL/6J mice. Diabetes.

[pgen-0020081-b013] Almind K, Kahn CR (2004). Genetic determinants of energy expenditure and insulin resistance in diet-induced obesity in mice. Diabetes.

[pgen-0020081-b014] Ahren B, Pacini G (2002). Insufficient islet compensation to insulin resistance versus reduced glucose effectiveness in glucose-intolerant mice. Am J Physiol.

[pgen-0020081-b015] Burcelin R, Crivelli V, Dacosta A, Roy-Tirelli A, Thorens B (2002). Heterogeneous metabolic adaptation of C57BL/6J mice to high-fat diet. Am J Physiol Endocrinol Metab.

[pgen-0020081-b016] Rossmeisl M, Rim JS, Koza RA, Kozak LP (2003). Variation in type 2 diabetes–related traits in mouse strains susceptible to diet-induced obesity. Diabetes.

[pgen-0020081-b017] Bassett DR, Craig BW (1988). Influence of early nutrition on growth and adipose tissue characteristics in male and female rats. J Appl Physiol.

[pgen-0020081-b018] Fiorotto ML, Burrin DG, Perez M, Reeds PJ (1991). Intake and use of milk nutrients by rat pups suckled in small, medium, or large litters. Am J Physiol.

[pgen-0020081-b019] Schmidt I, Schoelch C, Ziska T, Schneider D, Simon E (2000). Interaction of genetic and environmental programming of the leptin system and of obesity disposition. Physiol Genomics.

[pgen-0020081-b020] Leibel RL, Rosenbaum M, Hirsch J (1995). Changes in energy expenditure resulting from altered body weight. N Engl J Med.

[pgen-0020081-b021] Rosenbaum M, Vandenborne K, Goldsmith R, Simoneau JA, Heymsfield S (2003). Effects of experimental weight perturbation on skeletal muscle work efficiency in human subjects. Am J Physiol Regul Integr Comp Physiol.

[pgen-0020081-b022] Stock MJ (1999). Gluttony and thermogenesis revisited. Int J Obes Relat Metab Disord.

[pgen-0020081-b023] Tong Q, Dalgin G, Xu H, Ting CN, Leiden JM (2000). Function of GATA transcription factors in preadipocyte-adipocyte transition. Science.

[pgen-0020081-b024] Freytag SO, Geddes TJ (1992). Reciprocal regulation of adipogenesis by Myc and C/EBP alpha. Science.

[pgen-0020081-b025] Collins S, Daniel KW, Rohlfs EM, Ramkumart V, Taylor IL (1994). Impaired expression and functional activity of the β_3_- and β_1_-adrenergic receptors in adipose tissue of congenitally obese (C57BL/6J *ob/ob*) mice. Mol Endocrinol.

[pgen-0020081-b026] Ross SE, Hemati N, Longo KA, Bennett CN, Lucas PC (2000). Inhibition of adipogenesis by Wnt signaling. Science.

[pgen-0020081-b027] Bennett CN, Ross SE, Longo KA, Bajnok L, Hemati N (2002). Regulation of Wnt signaling during adipogenesis. J Biol Chem.

[pgen-0020081-b028] Rahmouni K, Mark AL, Haynes WG, Sigund CD (2004). Adipose depot-specific modulation of angiotensinogen gene expression in diet-induced obesity. Am J Physiol Endocrinol Metab.

[pgen-0020081-b029] Daluiski A, Engstrand T, Bahamonde ME, Gamer LW, Agius E (2001). Bone morphogenetic protein-3 is a negative regulator of bone density. Nat Genet.

[pgen-0020081-b030] Friedman JM (2003). A war on obesity, not the obese. Science.

[pgen-0020081-b031] Hill JO, Wyatt HR, Reed GW, Peters JC (2003). Obesity and the environment: Where do we go from here?. Science.

[pgen-0020081-b032] Barker DJ, Hales CN, Fall CH, Osmond C, Phipps K (1993). Type 2 (non-insulin-dependent) diabetes mellitus, hypertension, and hyperlipidaemia (syndrome X): Relation to reduced fetal growth. Diabetologia.

[pgen-0020081-b033] Armitage JA, Khan IY, Taylor PD, Nathanielsz PW, Poston L (2004). Developmental programming of the metabolic syndrome by maternal nutritional imbalance: How strong is the evidence from experimental models in mammals?. J Physiol.

[pgen-0020081-b034] Van Heek M, Compton DS, France CF, Tedesco RP, Fawzi AB (1997). Diet-induced obese mice develop peripheral, but not central, resistance to leptin. J Clin Invest.

[pgen-0020081-b035] Yura S, Itoh H, Sagawa N, Yamamoto H, Masuzaki H (2005). Role of premature leptin surge in obesity resulting from intrauterine undernutrition. Cell Metab.

[pgen-0020081-b036] Ailhaud G, Hauner H, Bray GA, Bouchard C (2004). Development of white adipose tissue.

[pgen-0020081-b037] Aoki N, Yamaguchi Y, Ohira S, Matsuda T (1999). High-fat feeding of lactating mice causing a drastic reduction in fat and energy content in milk without affecting the apparent growth of their pups and the production of major milk fat globule membrane components MFG-E8 and butyrophilin. Biosci Biotechnol Biochem.

[pgen-0020081-b038] MacDougald OA, Lane MD (1995). Transcriptional regulation of gene expression during adipocyte differentiation. Annu Rev Biochem.

[pgen-0020081-b039] Rosen ED, Spiegelman BM (2000). Molecular regulation of adipogenesis. Annu Rev Cell Dev Biol.

[pgen-0020081-b040] Bennett CN, Longo KA, Wright WS, Suva LJ, Lane TF (2005). Regulation of osteoblastogenesis and bone mass by Wnt10b. Proc Natl Acad Sci U S A.

[pgen-0020081-b041] Kennell JA, MacDougald OA (2005). Wnt signaling inhibits adipogenesis through beta-catenin-dependent and -independent mechanisms. J Biol Chem.

[pgen-0020081-b042] Logan CY, Nusse R (2004). The Wnt signaling pathway in development and disease. Annu Rev Cell Dev Biol.

[pgen-0020081-b043] Longo KA, Wright WS, Kang S, Gerin I, Chiang SH (2004). Wnt10b inhibits development of white and brown adipose tissues. J Biol Chem.

[pgen-0020081-b044] Spiegelman BM, Ginty CA (1983). Fibronectin modulation of cell shape and lipogenic gene expression in 3T3-adipocytes. Cell.

[pgen-0020081-b045] McBeath R, Pirone DM, Nelson CM, Bhadriraju K, Chen CS (2004). Cell shape, cytoskeletal tension, and RhoA regulate stem cell lineage commitment. Dev Cell.

[pgen-0020081-b046] Cheng SL, Shao JS, Charlton-Kachigian N, Loewy AP, Towler DA (2003). MSX2 promotes osteogenesis and suppresses adipogenic differentiation of multipotent mesenchymal progenitors. J Biol Chem.

[pgen-0020081-b047] Rupnick MA, Panigrahy D, Zhang CY, Dallabrida SM, Lowell BB (2002). Adipose tissue mass can be regulated through the vasculature. Proc Natl Acad Sci U S A.

[pgen-0020081-b048] Fukumura D, Ushiyama A, Duda DG, Xu L, Tam J (2003). Paracrine regulation of angiogenesis and adipocyte differentiation during in vivo adipogenesis. Circ Res.

[pgen-0020081-b049] Skurk T, Hauner H (2004). Obesity and impaired fibrinolysis: Role of adipose production of plasminogen activator inhibitor-1. Int J Obes Relat Metab Disord.

[pgen-0020081-b050] Ma LJ, Mao SL, Taylor KL, Kanjanabuch T, Guan Y (2004). Prevention of obesity and insulin resistance in mice lacking plasminogen activator inhibitor 1. Diabetes.

[pgen-0020081-b051] Schafer K, Fujisawa K, Konstantinides S, Loskutoff DJ (2001). Disruption of the plasminogen activator inhibitor 1 gene reduces the adiposity and improves the metabolic profile of genetically obese and diabetic ob/ob mice. Faseb J.

[pgen-0020081-b052] Shindo T, Kurihara H, Kuno K, Yokoyama H, Wada T (2000). ADAMTS-1: A metalloproteinase-disintegrin essential for normal growth, fertility, and organ morphology and function. J Clin Invest.

[pgen-0020081-b053] Selvarajan S, Lund LR, Takeuchi T, Craik CS, Werb Z (2001). A plasma kallikrein-dependent plasminogen cascade required for adipocyte differentiation. Nat Cell Biol.

[pgen-0020081-b054] Rakyan VK, Chong S, Champ ME, Cuthbert PC, Morgan HD (2003). Transgenerational inheritance of epigenetic states at the murine Axin(Fu) allele occurs after maternal and paternal transmission. Proc Natl Acad Sci U S A.

[pgen-0020081-b055] Koza RA, Hohmann SM, Guerra C, Rossmeisl M, Kozak LP (2000). Synergistic gene interactions control the induction of the mitochondrial uncoupling protein (Ucp1) gene in white fat tissue. J Biol Chem.

[pgen-0020081-b056] Coulter AA, Bearden CM, Liu X, Koza RA, Kozak LP (2003). Dietary fat interacts with QTLs controlling induction of Pgc-1 alpha and Ucp1 during conversion of white to brown fat. Physiol Genomics.

[pgen-0020081-b057] Thomas PD, Campbell MJ, Kejariwal A, Mi H, Karlak B (2003). PANTHER: A library of protein families and subfamilies indexed by function. Genome Res.

[pgen-0020081-b058] Ailhaud G, Teboul M, Massiera F (2002). Angiotensinogen, adipocyte differentiation, and fat mass enlargement. Curr Opin Clin Nutr Metab Care.

[pgen-0020081-b059] Voros G, Maquoi E, Collen D, Lijnen HR (2003). Differential expression of plasminogen activator inhibitor-1, tumor necrosis factor-alpha, TNF-alpha converting enzyme, and ADAMTS family members in murine fat territories. Biochim Biophys Acta.

[pgen-0020081-b060] Fruebis J, Tsao TS, Javorschi S, Ebbets-Reed D, Erickson MR (2001). Proteolytic cleavage product of 30-kDa adipocyte complement-related protein increases fatty acid oxidation in muscle and causes weight loss in mice. Proc Natl Acad Sci U S A.

[pgen-0020081-b061] Kishida K, Kuriyama H, Funahashi T, Shimomura I, Kihara S (2000). Aquaporin adipose, a putative glycerol channel in adipocytes. J Biol Chem.

[pgen-0020081-b062] Otto TC, Lane MD, Cox MM (2005). Adipose development: From stem cell to adipocyte. Crit Rev Biochem Mol Biol.

[pgen-0020081-b063] Castro CH, Shin CS, Stains JP, Cheng SL, Sheikh S (2004). Targeted expression of a dominant-negative N-cadherin in vivo delays peak bone mass and increases adipogenesis. J Cell Sci.

[pgen-0020081-b064] Whitley P, Gibbard AM, Koumanov F, Oldfield S, Kilgour EE (2002). Identification of centaurin-alpha2: A phosphatidylinositide-binding protein present in fat, heart, and skeletal muscle. Eur J Cell Biol.

[pgen-0020081-b065] Hikasa H, Sokol SY (2004). The involvement of Frodo in TCF-dependent signaling and neural tissue development. Development.

[pgen-0020081-b066] Kawano Y, Kypta R (2003). Secreted antagonists of the Wnt signalling pathway. J Cell Sci.

[pgen-0020081-b067] Hutley L, Shurety W, Newell F, McGeary R, Pelton N (2004). Fibroblast growth factor 1: A key regulator of human adipogenesis. Diabetes.

[pgen-0020081-b068] Jones BK, Levorse J, Tilghman SM (2001). Deletion of a nuclease-sensitive region between the Igf2 and H19 genes leads to Igf2 misregulation and increased adiposity. Hum Mol Genet.

[pgen-0020081-b069] Drevon CA (2005). Fatty acids and expression of adipokines. Biochim Biophys Acta.

[pgen-0020081-b070] Westendorf JJ, Kahler RA, Schroeder TM (2004). Wnt signaling in osteoblasts and bone diseases. Gene.

[pgen-0020081-b071] Phan J, Peterfy M, Reue K (2004). Lipin expression preceding peroxisome proliferator-activated receptor-gamma is critical for adipogenesis in vivo and in vitro. J Biol Chem.

[pgen-0020081-b072] Pinson KI, Brennan J, Monkley S, Avery BJ, Skarnes WC (2000). An LDL-receptor-related protein mediates Wnt signalling in mice. Nature.

[pgen-0020081-b073] Takahashi M, Kamei Y, Ezaki O (2005). Mest/Peg1 imprinted gene enlarges adipocytes and is a marker of adipocyte size. Am J Physiol Endocrinol Metab.

[pgen-0020081-b074] Yan D, Wallingford JB, Sun TQ, Nelson AM, Sakanaka C (2001). Cell autonomous regulation of multiple Dishevelled-dependent pathways by mammalian Nkd. Proc Natl Acad Sci U S A.

[pgen-0020081-b075] Viguerie N, Vidal H, Arner P, Holst C, Verdich C (2005). Adipose tissue gene expression in obese subjects during low-fat and high-fat hypocaloric diets. Diabetologia.

[pgen-0020081-b076] Kang YJ, Jeon ES, Song HY, Woo JS, Jung JS (2005). Role of c-Jun N-terminal kinase in the PDGF-induced proliferation and migration of human adipose tissue-derived mesenchymal stem cells. J Cell Biochem.

[pgen-0020081-b077] Saltiel AR, Kahn CR (2001). Insulin signalling and the regulation of glucose and lipid metabolism. Nature.

[pgen-0020081-b078] Nigg EA, Eppenberger HM, Jans DA, Hemmings BA, Hilz H, Cullen B, Gage LP, Siddiqui MAQ, Skalka AM, Weissbach H (1988). Evidence for transcriptional activation of the plasminogen activator gene by the catalytic subunit of cAMP-dependant protein kinase. Mechanisms of control of gene expression.

[pgen-0020081-b079] Johnson GB, Riggs BL, Platt JL (2004). A genetic basis for the “Adonis” phenotype of low adiposity and strong bones. Faseb J.

