# Supplementary material for: Changes in Gene Expression Foreshadow Diet-Induced Obesity in Genetically Identical Mice
Source: PLoS Genet. 2006 May 26;2(5):e81. doi: 10.1371/journal.pgen.0020081 (PMC1464831; doi:10.1371/journal.pgen.0020081)
Supplement: Table S1 — (131 KB PDF) [file pgen.0020081.st001.pdf]

Supplement Table 1. Microarray Analysis of the Hypothalamus of High and Low Gainer Mice

		Ratio high gainers/low gainers					
		**Microarray**		**QRT-PCR, normalized to cyclophilin**			
**Microarray genes**		pooled samples[a]	p value	pooled samples[b]	p value	individual samples[c]	p value
POMC	pro-opiomelanocortin-α	2.836	0.000	6.907	0.001	1.258[d]	0.201
PITP	Phosphatidylinositol transfer protein	1.539	0.004	1.106	0.197	1.120	0.284
Riken	RIKEN cDNA 1110020B03 gene	1.577	0.005	0.976	0.234	0.932	0.031
HCN2	hyperpolarization-activated, cyclic nucleotide-gated K+ 2	2.360	0.009	1.079	0.338	1.114	0.044
CAP	adenylate cyclase-associated protein 1	2.184	0.009	0.991	0.162	1.138	0.054
Renin	Renin	1.499	0.003	1.202	0.032	0.977	0.330
GAPDH3	glyceraldehyde-3-phosphate dehydrogenase	1.453	0.004	1.081	0.146	0.951	0.147
Gγ2	G protein γ2	0.800	0.008	1.301	0.063	0.985	0.458
**Hypothalamic satiety-related genes**							
AGRP	agouti-related peptide	1.530	0.661	0.912	0.249	0.724	0.286
GABA-A Rα1	GABA-A receptor, subunit α 1	1.287	0.553	0.939	0.475	1.005	0.449
GABA-A Rα3	GABA-A receptor, subunit α 3	0.640	0.568	1.252	0.480	1.060	0.064
Gαs	G protein, αs	1.013	0.543	0.950	0.196	1.005	0.295
Lep-R	leptin receptor	0.769	0.996	0.853	0.391	1.140	0.046
STAT3	Signal transducer and activator of transcription 3	0.495	0.283	0.999	0.123	1.026	0.265
MC3R	melanocortin 3 receptor	1.218	0.537	1.202	0.244	0.963	0.281
MC4R	melanocortin 4 receptor	0.531	0.815	0.992	0.465	1.023	0.297
NPY	neuropeptide Y	0.802	0.192	1.106	0.475	0.972	0.384
NPYR2	neuropeptide Y receptor 2	0.904	0.363	0.933	0.171	0.956	0.117

[a] Pooled samples constituted 20 low or 20 high gainers; 4 microarray chips for each pool.
[b] Pooled samples were assayed by QRT-PCR in quadruplicate.
[c] QRT-PCR was performed on each of the 20 low and 20 high gainers in duplicate.
[d] Single outlier removed from analysis
